# Patient and Provider Demand for and Readiness to Adopt Medications for Stimulant Use Disorders in Real‐World Settings: Pre‐Planning Toward Accelerated Implementation

**DOI:** 10.1111/dar.70247

**Published:** 2026-09-16

**Authors:** Enya B. Vroom, Erin P. Finley, Carma Deem Bolton, Tara Karns‐Wright, Jennifer S. Potter

**Affiliations:** ^1^ Center for Research to Advance Community Health, Long School of Medicine The University of Texas Health Science Center at San Antonio San Antonio Texas USA; ^2^ Be Well Institute on Substance Use and Related Disorders, Long School of Medicine, The University of Texas Health Science Center at San Antonio San Antonio Texas USA; ^3^ Department of Medicine Long School of Medicine, The University of Texas Health Science Center at San Antonio San Antonio Texas USA; ^4^ Department of Psychiatry and Behavioral Sciences Long School of Medicine, The University of Texas Health Science Center at San Antonio San Antonio Texas USA; ^5^ Center for the Study of Healthcare Innovation, Implementation, and Policy, Veterans Affairs Greater Los Angeles Healthcare System Los Angeles California USA

**Keywords:** adoption, barriers and facilitators, contextual factors, lived experience, medication for stimulant use disorder, qualitative

## Abstract

**Introduction:**

In the United States, rates of stimulant use disorder (StUD) have increased over the last 20 years, yet fewer than one‐third of adults receive treatment. Recent research has shown the efficacy of medication for StUD, but little is known regarding demand and readiness to adopt such treatments. This study examined patient and provider perspectives on medication for StUD in real‐world practice settings.

**Methods:**

Semi‐structured interviews were conducted with patients with substance use disorder (SUD) history (*n* = 20) and SUD care team members (*n* = 20) currently providing SUD services. Interviews elicited perceptions of treatment demand, barriers/facilitators, and recommendations for future implementation. Rapid qualitative analysis was used to distill and compare findings across groups. Analysis was guided by the Health Equity Implementation Framework (HEIF), selected for its focus on implementation and social determinants.

**Results:**

Consistent with HEIF, multilevel patient and care team barriers and facilitators were identified, reflecting the importance of the clinical encounter and highlighting implementation challenges within the inner, outer and societal contexts, particularly related to stigma. Training for providers and education for patients and families were recommended to overcome stigma. Utilising patient‐centred care approaches and addressing societal factors impacting treatment engagement were also discussed as likely to affect medication access.

**Discussion and Conclusions:**

Examining patient and care teams' perceptions of medications for StUD improves understanding of demand and readiness for adoption. This information, rooted in lived experiences, provides a foundation for selecting tailored implementation strategies that may assist in accelerating the adoption of medications to treat StUD in future scale‐up and spread.

## 1 Introduction

Globally, stimulants (i.e., cocaine, prescription stimulants or methamphetamine) are the second most used controlled substance [1]. In the United States (US), recent overdose trends show stimulants combined with opioids account for a large majority of overdose fatalities [2], and there was a 61% increase in methamphetamine‐related overdose deaths between 1999 and 2021 [3]. Despite significant morbidity and mortality, rates of stimulant use disorder (StUD) treatment remain low globally [1, 4, 5]. There is growing evidence supporting the clinical efficacy of medication for StUD; however, no medications are currently approved by the US Food and Drug Administration (FDA) or any other regulatory authority worldwide, which limits access to evidence‐based care [6].

Even interventions with decades of robust evidence, such as contingency management for cocaine and methamphetamine use disorders, are still underutilised in routine practice due to stigma (e.g., seen as rewarding drug use), cost and significant implementation challenges [7, 8]. Given these barriers, pharmacotherapies may have a greater likelihood of utilisation, particularly in settings where stigma is high and the policy environment is not supportive of contingency management [9]. Recent studies show evidence for the clinical efficacy of a naltrexone plus bupropion combination and other monotherapies such as mirtazapine in treating methamphetamine use disorder [10, 11, 12, 13]. Both non‐psychostimulant (e.g., bupropion) and psychostimulant (e.g., amphetamine salts) medications are being increasingly used ‘off‐label’ to treat StUD globally, and in the US, are now recommended in the American Society of Addiction Medicine/American Academy of Addiction Psychiatry's clinical practice guideline for StUD [14]. In 2023, the US FDA published guidance to encourage the development and evaluation of StUD medications [15]. As evidence of the efficacy and effectiveness of StUD medication grows globally, it is critical to examine contextual factors that influence implementation across the translational science spectrum (i.e., T1‐T4) to accelerate service delivery [16]. Although there is an increasing body of evidence on strategies for medications for opioid use disorder (MOUD; [17, 18, 19]), this is the first study of which we are aware to assess readiness to adopt medications for StUD using a multilevel implementation science framework incorporating both patient and provider perspectives.

Despite the recent success of clinical trials, there has been limited focus on understanding best practices and challenges in delivering medication for StUD treatment [20]. Responding to calls for the integration of implementation science into efforts to increase the availability of evidence‐based therapies for substance use disorders (SUD; [21, 22]), we drew upon the Health Equity Implementation Framework (HEIF) to explore barriers and facilitators likely to influence the adoption of medication for StUD in SUD treatment settings [23, 24]. Responding to literature highlighting decades of stigma and negative clinical encounters faced by individuals who use stimulants [20, 25], we selected the HEIF due to its focus on implementation determinants not only within clinical practice settings, but also in broader contexts such as healthcare systems and society. The HEIF identifies factors across multiple contextual levels, including: (i) the innovation (medication for StUD); (ii) recipients (care teams and patients); (iii) the clinical encounter; (iv) the inner context (organisations where SUD treatment occurs [e.g., primary care, specialty care, emergency departments]); (v) the outer context (healthcare system); and (vi) the societal context (economies, physical structures and sociopolitical factors) [23, 24]. Consensus on the nature of treatment and recovery for StUD is crucial in creating and implementing successful solutions for patients [9]. By incorporating insights from the lived experiences of patients and SUD care teams and lessons learned from MOUD implementation research, we aim to enhance understanding of the readiness to adopt such treatments and strategies needed for effective scale‐up and implementation in real‐world practice.

## 2 Methods

### 2.1 Participants

Participants included patients with current or a history of SUD (*n* = 20) and members of SUD care teams, including prescribing providers and other care team professionals (e.g., clinical directors), who currently treat patients with SUD (*n* = 20). The study used purposive and snowball sampling to identify and recruit patient and care team participants from substance use treatment settings within Texas (*n* = 35), Pennsylvania (*n* = 2), Louisiana (*n* = 1) and Illinois (*n* = 1). One participant did not disclose their location (*n* = 1).

Care team recruitment involved distributing study information through the authors' institutional listservs, including an established network of over 80 substance use treatment organisations in Texas, and attendees of harm reduction, hospital opioid use disorder treatment, and medications for substance use disorders Extension for Community Healthcare Outcomes series (> 500 registrants nationwide). Care team members included chief executive officers (*n* = 2), clinic directors (*n* = 5), counsellors (*n* = 3), physicians (*n* = 7) and nurse practitioners (*n* = 2) employed by organisations providing SUD treatment in outpatient and inpatient settings, including addiction specialty care, primary care, and integrated mental health and addiction care. One counsellor also identified as a certified peer support specialist (*n* = 1). We intentionally sampled multidisciplinary care team members, including prescribing and non‐prescribing providers, to capture a broad range of perspectives relevant to readiness and adoption.

Patient recruitment involved distributing study information through care members' organisations and local contacts, as well as the same institutional listservs used for care team recruitment, where care team members were asked to share study information with their patients. Existing patients and care team participants also referred others to the study. To capture a broad range of perspectives among patients with experience with or direct exposure to stimulants, to account for polysubstance use, and to overcome recruitment barriers among a historically hard‐to‐reach population (related to high stigma), we did not require a formal StUD diagnosis for inclusion. As a result, patient participants were eligible to participate if they had a current or prior SUD.

This study was reviewed and approved by the University of Texas Health Science Center at San Antonio's Institutional Review Board. All participants received a $50 gift card for their participation.

### 2.2 Data Collection

Virtual interviews took place between December 2023 and April 2024, were recorded with participant consent, and were stored on a virtual *Health Insurance Portability and Accountability Act (HIPAA)*‐compliant platform. Interviews lasted approximately 45 min and were conducted by three research analysts who were trained by the co‐principal investigators (EBV, EPF), both of whom have significant experience in qualitative research methodologies. The study developed and used a semi‐structured interview guide informed by the HEIF. Patient and care team interview guides included open‐ended questions to elicit participant perspectives on: (i) StUD treatment demand; (ii) social influences on treatment engagement/retention; (iii) StUD‐focused clinical encounters; (iv) care team, patient and community knowledge/attitudes to StUD treatment; (v) barriers/facilitators to adopting medications for StUD, including perceptions of a specific medication combination for StUD that has demonstrated clinical efficacy (i.e., injectable naltrexone and oral bupropion) [12, 13]; and (vi) recommendations to support future adoption and implementation. To facilitate the comparison of responses across groups, patient and care team interview guides covered the same overarching topics, with slight variations in the wording of questions to ensure clarity and accessibility [26].

### 2.3 Data Analysis

Guided by the Planning for and Assessing Rigor in Rapid Qualitative Analysis framework [27], we used a rapid qualitative approach to analyse interview data. This approach is relevant for the current study as it can efficiently transform findings into actionable goals/contributions toward future implementation and/or research efforts [28, 29]. Shortly after the conclusion of each interview, interview transcriptions were reviewed for accuracy and analysts conducting the interviews created a structured summary for each, following a summary template of topic domains aligned with key HEIF constructs (e.g., innovation, recipients, clinical encounter, inner and outer context, and societal context) and allowing for the capture of emergent findings. The research team met weekly during data collection and analysis to ensure consistency in processes and approach. Additionally, the study's co‐PI (EBV) reviewed all summaries for consistency and completeness. Summary content was then distilled into a matrix structured by respondent and topic domain, allowing for a comprehensive review of findings. The team then met periodically over five weeks to summarise data related to each topic domain into a narrative summary; these were discussed and refined, referring constantly back to the matrix (and, where necessary, original transcripts) for verification and to enhance the validity of synthesised findings [27]. Once narrative summaries were completed, the team reviewed and compared findings within and across patient and care team samples, critically reviewed findings for alignment with the HEIF, and established consensus on key findings within each HEIF domain.

## 3 Results

**FIGURE 1:**
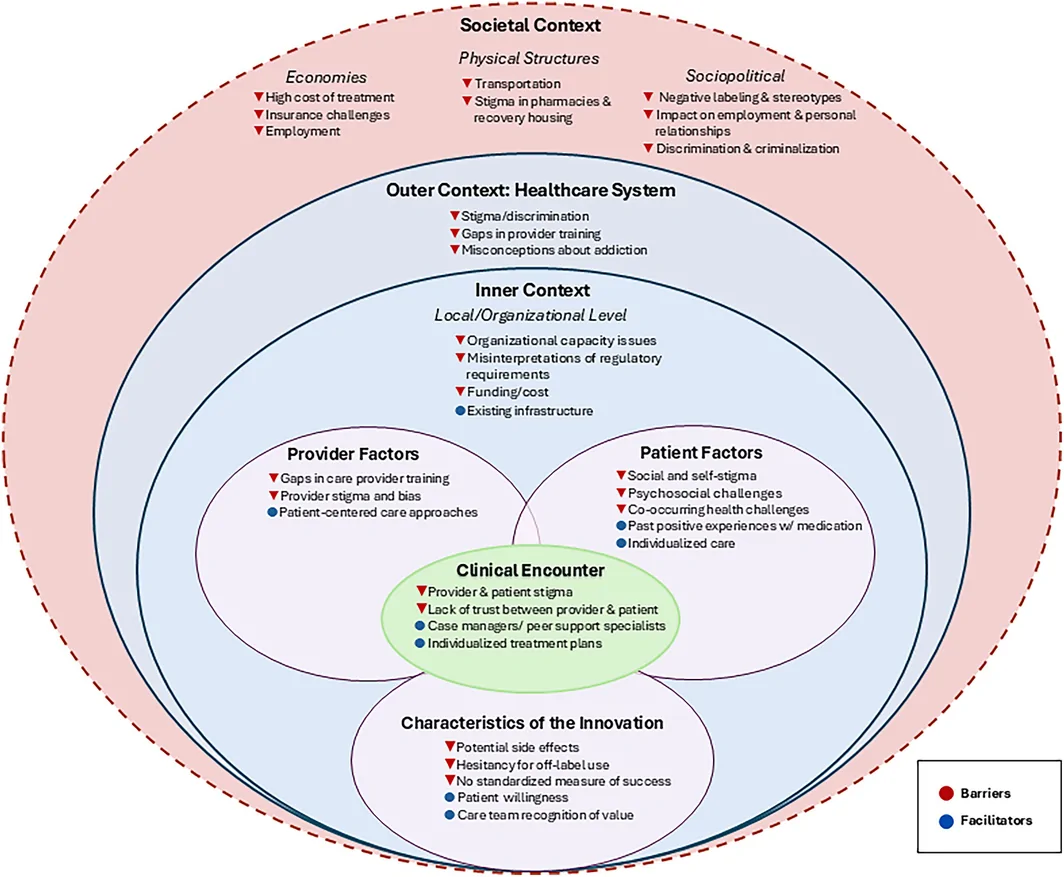
Patient and provider perspectives on medication for stimulant use disorder within the Health Equity Implementation Framework.

In total, 40 interviews were conducted with patients (*n* = 20) and SUD care team members (*n* = 20). The findings below are presented by HEIF domains, highlighting their relationship to the adoption and delivery of medication for StUD in real‐world practice. Several key findings, including stigma, care provider training, and patient‐centred care, are present across multiple HEIF domains. Figure 1 outlines key findings across all HEIF domains, and Table 1 includes representative quotes.

**TABLE 1:** Representative quotes from patient and care team interviews.

HEIF domains	Representative quotes	
	Facilitators	Barriers
Characteristics of the Innovation	Patient support of StUD medication options: ‘*… it would be really helpful if there was some sort of medication‐assisted treatment like there is for opioids. There just isn't. And in my experience, people relapse over and over until they either finally get over it or die.’*—Patient 133	Patient StUD medication hesitancy: *‘… it's a lot of these newer medicines that come out, it's hard to [trust] just because the testing time in them is relatively short, and it seems like a lot of the newer medications they're using, it's like off‐label use. … it's not necessarily what they were intended for. That always makes me a little bit weary, because it just feels like they're just throwing darts at the wall and seeing what will stick*.’—Patient 132 Care provider concerns about no standardised measures of success for StUD medication: *‘… we don't have standard measures of success in the same way we do with opioid use disorder [OUD] in terms of stimulant use disorder. … I wish I could tell you a better answer based on the kind of agreed‐upon key performance indicators against a benchmark similar to how we can do retention in treatment for OUD*.’—Care provider 105
Recipient Factors *Patient Factors*	Patient familiarity with medication could positively impact willingness to engage: *‘So, I know about naltrexone. Very well. And then … I used to take Wellbutrin [bupropion] for about 6 to 7 months or so. … let's say you're taking Wellbutrin every day. If that's something that you're able to get a month's supply of at a time, or even just a three‐week supply whenever you would go and get your Naltrexone shot, that would be very helpful … it sounds like a perfect combination*.’—Patient 179	Stigma from family affecting patient treatment engagement: ‘*Families will disown their family members that are actually using and not help them, desert them. … But that actually does more harm than good. …say your child was, you know, in college and started using drugs. Okay, you cut them off. But if your child is in college and they're having a hard time, you'll still feed them, right? So why not continue to help feed them?*’—Care provider 109 Patients' unmet basic needs hindering treatment engagement: ‘*What we see is people struggling with basic needs first. So, do they have a place to sleep and live? Do they have food? You know, just the basic safety/shelter needs. And many times, those supersede the actual treatment*.’—Care provider 111
*Care Team Factors*	—	Limited physician training fostering stigma/bias in patient care: *‘Most of my colleagues, and me, too, like we didn't learn this stuff in health professional school. And so there's a huge education and workforce gap that our patients are facing.’*—Care provider 102
Clinical Encounter	Highlighting the importance of individualised care and recovery: *‘Everybody's recovery is different. They've got life coaches, they've got recovery coaches … when my daughter was in school, they had IEPs* … *you know, education plans. [If] we could have … like a team that everybody's able to do like individual recovery plans. Follow somebody through, like a whole recovery team, have CPS sit and meet with somebody … and then have the medical team* … *If we could just have an individualised program for people.’*—Patient 127	Treatment‐seeking experience shaped by patient's culture: *‘Stigma is the first thing that keeps you from speaking of it [history of SUD]* … *seeking help makes you feel like you have your shit [out in] front of everybody* … *for a person like me it's a very difficult position. … I think it's a part of Black culture to just want to be respected because of the way you'd be treated, especially someone who is using again* … *Even if you do start [treatment], you'd want to quit immediately* … *t's uncomfortable …’*—Patient 121 Patient perspective on lack of provider empathy: *‘I feel like some providers should have more heart when it comes to addicts because they could save a life. … being more empathetic … I was actually pregnant at the time. And I went to a hospital and they gave me ibuprofen, told me to wait in the lobby, and I'm like, “no, I really need some help, like I have nowhere to go. I'm a drug addict,” and they were just like ‘sleep it off.’’*—Patient 124
Inner Context	Treatment organisations' familiarity with medication treatments can assist with buy‐in and adoption: *“So, we already have and store Naltrexone … we use stock of it all the time. We have a program that allows us to give it to clients before they even leave. You know, injections … We would kinda [set up medication for StUD treatment] the way we do it now.”*—Care provider 120	Treatment organisations having limited staff capacity: *‘So, for a lot of mental health facilities, it's usually a lack of staff. That's number one. … there is somewhat of a turnover, which, of course, directly trickles down into the treatment of participants’*.—Care provider 107 Organisational considerations of risk and regulations: *‘Definitely spending some time training the treatment facilities, the therapist, even at the CEO level. Because I would want to know as a CEO, ‘what is the risk for my organisation? What is the liability for my organisation?’ And knowing, “here's a risk. Here's a liability. But here are the systems in place to protect your agency, because I have to answer the board.” … and then I have to look at insurance costs and the bandwidth of training other people that work for the organisation.’*—Care provider 108 Previous experience with past SUD medication policies causing organisational hesitancy for future adoption: *‘… with the [X] waiver and everything before. I think a lot of providers I talked to don't know that that's no longer there, if they're not tuned into it. It's just like a little snippet of news that they miss. They're like, “oh, what we don't have to do that anymore?” I think the administrative regulatory barriers were a lot for a while, so everyone's just kind of like, “Whoa, I'm not doing that. I don't know how to do that.”’*—Care provider 114
Outer Context	—	Bias within the healthcare system: *‘Every patient I see has a reasonable expectation of mistreatment in the health care setting … So, there's structural stigma, there's institutionalised ignorance, and there's internalised stigma around patients’*.—Care provider 113
Societal Context *Economies*	—	Insurance delays impacting treatment wait times and patient engagement: *‘I had to delay going back into treatment because of my insurance. They said that I had to wait until after a certain amount of time, because that was their policy when it came to substance abuse treatment. And I mean, even just waiting an extra day whenever you're out there using could be deadly. You never know if the day that you should have gone into treatment is the day that you don't make it’*.—Patient 130 State or county‐funded programs, including Medicaid, coupled with patient social determinants of health, affect patient treatment access: *‘We've had a lot of people turned off of Medicaid with the recent Medicaid issues and limited access to commercial insurance. And then for patients who are on the county health plan or the hospital health district plan, they're typically ones with disproportionate social determinants, and they're also the group of people who don't have access to higher levels of addiction care because the system doesn't exist in most parts of [the state]’*.—Care provider 105 Coverage issues for injectable medications: *‘You know, if it's more long‐acting injectable … Naltrexone … very unlikely because we take care of poor people and there's no support from the state to do that … I think the reality here in [state] where we didn't expand Medicaid is that …'*—Care provider 115
*Physical Structures*	—	Treatment access challenges for patients in rural areas: *‘Most of my travel was about 60 miles from here to [nearest city], and I live in [rural town]. It's about an hour and 15 min to get over there. I wouldn't want to do that every day’*.—Patient 144 *‘So we see patients all over [the state] and a lot of them are very geographically isolated. So, getting to a lab, being able to find the pharmacy that carries their suboxone, and is not gonna discriminate against them picking it up, sometimes it is a big challenge’*.—Care provider 114
*Sociopolitical Factors*	—	Trickle‐down of societal‐level stigma through service systems: *‘Laziness. Stigma. Fear. Discrimination. Those are the main things on the part of the medical community* … *I just think most doctors are like most people, and don't stop to examine their motives or their judgements about people who use drugs’*.—Care provider 111 *‘[Patients are] being denied certain services*. … *Because at first, no [recovery homes] would take people who were on medication. So back in 2011, I made a choice to accept people who had to take medication, whether it was for their mental health, or to assist them with any other disorder. And I still see like the whole house was stigmatised and the whole program was stigmatised as if we are allowing people to “stay sick”’*.—Care provider 101 *‘The fact that addiction is criminalised instead of being treated as a health issue. A lot of people spend a lot of time being incarcerated instead of treated, and while that may force you to go cold‐turkey and get it out of your system, the chances are you're just waiting to get out so you can get started again, so that's a barrier’*.—Patient 122

### 3.1 Characteristics of the Innovation

Within the HEIF, characteristics of the innovation (i.e., medication for StUD) include the unique mechanisms or activities of an intervention that dictate how it will be applied in specific settings. Participants identified several innovation‐specific facilitators to adoption, such as patient willingness to try medication and care providers' recognition of its value to their patients, given the high prevalence of stimulant use in their communities, with many reporting that 50%–75% of their patients have a StUD. One care provider emphasised this need, stating, ‘I see [StUD] every day … if [medications] were available and partnered, it would make a huge impact’. Regarding the injectable naltrexone + bupropion combination, many patients were already familiar with bupropion, and care team members believed this familiarity would increase patients' willingness to consider this treatment option. The extended dosing schedule of injectable naltrexone (every three weeks) was also viewed favourably, potentially reducing the burden of frequent visits and improving adherence, particularly in the context of common barriers (e.g., lack of reliable transportation; childcare) that have proved challenging in the case of other SUD medications requiring more frequent visits (e.g., methadone).

The most frequently discussed barriers to adopting medication for StUD included concerns about side effects and potential interactions with other medications (e.g., benzodiazepines). Care teams expressed hesitancy in transitioning patients from buprenorphine or methadone to naltrexone due to concerns about detoxification and/or reducing patient buy‐in. Care teams also reported they would need more information on the scientific evidence of efficacy and effectiveness to feel comfortable adopting medication for StUD. Similarly, patients also wanted to better understand success rates and impact before considering medication as a treatment option: ‘Knowing the success rate of it, and if other people have used it … the effectiveness of it, knowing how it affects the body I think would be the important things for if I'd would take it or if I wouldn't’.

Most care team members were aware that there are no FDA‐approved medications to treat StUD. Some viewed the ability to use medications (e.g., naltrexone or bupropion) off‐label as an opportunity to expand treatment options for patients and saw naltrexone as easier to prescribe because it is not a controlled substance. However, participants' perspectives on off‐label use varied. While some patients expressed comfort with these medications, having been prescribed them for other conditions in the past, others reported hesitation, including discomfort with the idea of being a ‘test subject’, as one patient described, ‘I'm not going to be a guinea pig’. Some care providers reported that they would not feel comfortable prescribing in the absence of formal FDA approval. Lastly, care teams reported that there is no standardised measure of success for StUD treatment, which makes it challenging to ensure consistency and effectiveness.

### 3.2 Recipient Factors—Patients and Care Providers

Within HEIF, recipients are defined as individuals or groups who directly impact and/or are impacted by the implementation of an innovation. Patient recipients (who receive the innovation) and care team recipients (who deliver the innovation) bring their characteristics and experiences (e.g., demographics or bias) that may also influence implementation.

#### 3.2.1 Patient Factors

Patients shared that previous positive experiences with medication‐based treatment for other SUDs (e.g., OUD) influenced their openness to medication for StUD. In contrast, care teams discussed that patients may be less familiar with and trusting of the naltrexone + bupropion combination compared to more established treatments (e.g., buprenorphine), which could impact their willingness to engage in treatment, highlighting the importance of patient education.

In relation to barriers, patients most commonly reported internalised self‐stigma and external stigma from family, friends and/or community, both during active substance use and when seeking treatment. Many patients felt their families did not understand addiction as a chronic disease, leading to feelings of judgement and lack of support, which reduced their willingness to engage in treatment. Participants also highlighted the psychosocial challenges patients face when seeking treatment, including feelings of vulnerability, unclear treatment expectations, engaging in social settings/scenarios where drug use is prevalent, social isolation, and the need to leave family or work responsibilities to attend treatment. Care teams reported that many patients struggle with finding and maintaining the motivation to engage in treatment and recovery, often due to co‐occurring health challenges. For example, participants shared that a patient's unmet basic (e.g., housing), mental health (e.g., psychosis, post‐traumatic stress) or physical health needs (e.g., sleep, diabetes) can further hinder treatment engagement.

#### 3.2.2 Care Provider Factors

While patients did not identify any specific facilitators related to how care providers might increase delivery of medications for StUD, they agreed that well‐trained care teams and patient‐centerd care are crucial for treatment engagement. Both patients and care teams expressed concern that a lack of education in addiction medicine and medication treatment options could foster stigma and bias, negatively impacting clinical encounters. Patients and care teams emphasised the importance of training care providers about medication options for StUD, patient‐centred care approaches, and pathways of recovery (e.g., harm reduction) to reduce stigma and improve the quality of care.

### 3.3 Clinical Encounter

The clinical encounter refers to the patient–provider interaction, which plays a key role in treatment engagement, as it involves discussing shared and personal goals related to care. Patients and care teams reported a sense that stigma, exacerbated by inadequate training, could reduce providers' empathy in the clinical encounter. Lack of empathy was felt to be a significant barrier to patients seeking and engaging in medication treatment, particularly for underrepresented minority and/or pregnant patients.

Both patients and care teams emphasised the importance of trust in the patient–provider relationship, acknowledging that patients may be hesitant to disclose sensitive information (e.g., drug use) to a provider without first establishing rapport. Patients also highlighted the valuable role case managers or peer support specialists may play in increasing patient willingness to seek and engage in treatment. These care team members can serve as trusted sources of information, helping patients to navigate concerns about medication side effects, detox and weaning options, medication schedules, routes of administration, co‐occurring mental health disorders and other holistic care needs, including physical and spiritual well‐being. If case management is unavailable, patients emphasised the importance of care providers ensuring patients have the necessary information to make informed decisions about their treatment plan.

Beyond case management, both patients and care teams highlighted that treatment plans that address the whole person, including essential needs (e.g., housing) and personal preferences (e.g., faith‐based), are critical in fostering willingness to engage in treatment. Patients recommended that care teams receive training on viewing recovery as a journey to health, which may include medication treatment rather than solely focusing on abstinence. Recovery‐focused approaches were also recommended for reducing stigma and improving patient support. Additionally, patients noted that medication should be combined with therapy (group or individual) or peer support, sequenced before or during medication administration, to better address psychosocial needs (e.g., social support, mental health).

### 3.4 Inner Context

The inner context is comprised of local (e.g., clinic) and organisational (i.e., system in which the clinic is embedded) factors that may impact adoption and implementation. Local and organisational‐level factors can include organisational structures or policies, as well as local/state‐level regulation of medication for StUD.

Patients discussed inadequately equipped SUD treatment organisations as a significant barrier to treatment. For example, many inpatient clinics lack enough beds to meet patient volume and demand, resulting in long wait times for treatment. Limited outpatient appointment availability, especially during standard work hours (9:00 AM–5:00 PM), can make it difficult for those with traditional work schedules to attend treatment or make injection appointments (naltrexone‐specific). Patients also reported the importance of treatment organisations having the necessary resources for or partnerships with wraparound services such as recovery housing and childcare, which represent comprehensive, holistic care structures. Additionally, a lack of reliable transportation was frequently cited as a major obstacle to receiving care. Patients noted that treatment organisations offering transportation assistance (e.g., bus passes) or providing telehealth options for screening or check‐ins had a positive impact on their ability to engage in addiction treatment generally.

Care teams discussed how the quality of available treatments may be affected by high staff turnover and noted that securing and managing organisational funding can affect their ability to offer medication, ultimately determining how many patients can receive care and for what duration. A common concern was the lack of comprehensive services, which both patients and care teams attributed to insufficient organisational funding and resource access. Care teams also expressed a need for a clearer understanding of the logistical and legal implications of offering medication for StUD, recommending more information on training and licensing requirements, contracting with pharmacies and/or maintaining medication on‐site, securing organisational administrative support, and navigating cost or external funding requirements. Care teams believed that misinterpretations or a lack of awareness of current regulations and past experience with burdensome regulatory requirements for other SUD medications could create hesitancy among care providers and treatment organisations regarding the adopting of medication for StUD.

Facilitating the likelihood of medication uptake, care teams suggested that treatment organisations with prior experience in providing medication for other SUDs may be more confident in adopting similar treatment for StUD due to their established infrastructure (e.g., storage, workflows) and familiarity with implementation.

### 3.5 Outer Context

The outer context includes healthcare system factors such as broader policy drivers, perceptions, and mandates that can influence implementation and other HEIF domains, such as the clinical encounter and the inner context. Both patients and care teams revisited the concept of stigma as a larger issue within healthcare systems, preventing patients from seeking, engaging and adhering to treatment. Participants discussed how inadequate care provider training on addiction, especially for physicians, may foster discriminatory practices, such as denying medical services to patients or inconsistencies in screening procedures. Participants reported that some care providers can reinforce and perpetuate the misconception that medication for SUD is merely ‘trading one illicit drug for another’. Notably, neither patients nor care teams identified any facilitators related to SUD or StUD treatment in the broader healthcare system.

### 3.6 Societal Context

HEIF further distinguishes societal context, including economies, physical structures, and sociopolitical forces, as factors outside of the healthcare system that may influence all other HEIF domains. Economies describe the current state of wealth and resources of individuals seeking services (e.g., health insurance). Physical structures represent the physical environment where the services may be located. Sociopolitical forces involve formal or informal structures, policies and local culture that either promote or hinder services (e.g., laws, discrimination). Within the societal context domain, most of the related findings were described as barriers.

#### 3.6.1 Economies

Patients and care teams reported a lack of health insurance as a major barrier to accessing medication treatment due to the high cost, especially for injectable medications like naltrexone, which can exceed $1000 per month for uninsured individuals. Some patients discussed that even individuals with insurance may face challenges, including long wait times for treatment approval. Patients and care teams emphasised that most patients cannot afford treatment without insurance or access to state‐funded programs like Medicaid. This challenge was felt to be even greater in states that have not expanded Medicaid, which may further limit coverage options and extend wait times.

Patients also discussed employment‐related barriers, such as difficulties gaining or maintaining jobs due to drug screening, which can limit access to insurance (i.e., job benefits) and make paying for treatment out of pocket challenging. Additionally, patients reported that individuals with a history of SUD may engage in behaviours or actions that tarnish supportive relationships, lead to poor credit and further limit opportunities to obtain insurance (i.e., having a criminal record).

#### 3.6.2 Physical Structures

Limited access to reliable transportation was identified as a major physical barrier to treatment access and engagement, especially for those in rural areas. While resources like bus passes, Lyft credits or family support can help, they are not always available. Patients reported the physical location where an individual lives (e.g., rural or impoverished areas) may further impact treatment access and increase risk of drug use due to limited housing options and increased exposure to crime and drug availability. As one patient discussed, ‘When you live in like poverty‐stricken areas, you're not able to find good housing. You're gonna be exposed to a lot more crime and drugs’.

#### 3.6.3 Sociopolitical Factors

Patients and care teams discussed how stigma impacting perceptions of medication for StUD can be culturally rooted, such as the commonly perceived hierarchy of stimulants, with cocaine viewed less negatively than methamphetamines. Patients reported inaccurate and harmful portrayals of individuals who use stimulants in the media, as well as the use of stigmatising labels (e.g., ‘*crackhead*’), which further reinforce negative stereotypes. One patient discussed, ‘I think that that's kind of like, just the general understanding [of methamphetamine use], like nasty, bad, trashy’. Participants felt these negative perceptions can interact with other HEIF domains, raising the risk of internalised bias and discrimination in the clinical encounter. Care teams reported that deep‐rooted stigma can also contribute to discrimination in pharmacies, where medication may be perceived as ‘substituting one drug with another’, even resulting in refusals to fill prescriptions. Local stigma and preference for abstinence‐based approaches can result in recovery homes refusing to accept individuals receiving medication treatment. Additionally, patients reported that individuals with SUD, especially StUD, continue to be criminalised, resulting in more justice system involvement rather than having addiction recognised and addressed as a chronic health condition.

Both patients and care teams discussed the complexity of addiction and StUD, including the challenges created by co‐occurring mental health disorders and interpersonal problems, which participants felt were often misunderstood by the general public. Patients have felt others believe ‘their diagnosis is a choice’, which has limited their opportunities for employment and personal relationships (e.g., family, friends, or spouses). One patient shared a vulnerable reflection: ‘… so I feel like, people around wouldn't actually see us … you're a menace to society, you should not exist. They don't see you as a normal human being …’.

## 4 Discussion

The current study aimed to examine factors that can hinder or facilitate readiness for and the adoption of medication for StUD treatment in real‐world service settings. In inviting the perspectives and lived experiences of patients and SUD care teams, interviews elicited important considerations and recommendations for supporting effective scale‐up and spread. Utilising implementation science frameworks, like the HEIF, can aid in understanding how complex factors can occur across multiple contextual levels—from the individual to the organisation to broader social structures—and in strategising to overcome barriers that may otherwise hinder successful implementation [22]. The study highlights several key findings (see Figure 1) likely to impact broad implementation of medication for StUD, including stigma emerging across multiple HEIF domains, the importance of care team training in the evidence for and delivery of medication for StUD, the need for patient‐centred approaches to improve the clinical encounter and patient and family education to reduce stigma, and the likelihood that cost of medications, social determinants of health and limited insurance coverage will create barriers to treatment access, much as they have in the case of other medications for SUD treatment.

Stigma was identified as the most significant barrier across nearly all HEIF domains. Participants discussed that stigma may prevent patients from seeking, engaging in, and adhering to medication for StUD treatment. Historically, medication for SUDs has been largely stigmatised in the US [30]. Abstinence‐based care models hold the belief that medication treatment is still active use, which patients may internalise [31]. Internalised stigma may also negatively influence interpersonal relationships or be exacerbated by family and friends not accepting medications as valid medical treatments for SUD [32]. Bias within SUD treatment settings, the healthcare system, and broader society has led to perceptions of addiction being a moral choice as opposed to a chronic disease, which has affected patient access to services, quality of care, and overall recovery [32, 33]. Social stigma can also be seen through the grading of stimulants, with prescription stimulants and cocaine being more socially accepted than methamphetamines. The criminalisation of SUD, and more specifically StUD, has permeated the media, perpetuating stereotypes and derogatory labelling, as well as having impacted federal policy and funding allocation for harm reduction approaches [20].

The current study's findings indicate the need for targeted training and education of care teams, especially prescribing providers, on medication for StUD and best practices for clinical decision‐making. Some care team members expressed hesitancy in transitioning patients from opioid agonist therapy (e.g., buprenorphine) to naltrexone (an opioid antagonist) to treat co‐occurring OUD and StUD. This hesitancy is warranted, as current evidence does not support discontinuing agonist therapy in outpatient settings due to risks associated with the required opioid‐free period prior to naltrexone initiation [14]. When clinically indicated, transitions from agonist to antagonist should be undertaken with caution and under the supervision of an addiction medicine physician and/or a clinician with extensive experience in addiction treatment, consistent with American Society of Addiction Medicine guidelines. In cases where opioid agonist therapy is most appropriate, providers may also consider monotherapies, such as bupropion, mirtazapine or topiramate, which have shown some evidence of benefit for StUD [11, 34, 35, 36, 37]. These considerations underscore the importance of training in clinical guidance and decisions, particularly for providers in behavioural health or primary care settings who may have less knowledge or formal training in addiction medicine or psychiatry.

Additionally, incorporating stigma training into StUD treatment curricula for care teams that includes content to combat misconceptions and strategies for validating provider concerns has the potential to mitigate stigma in clinical encounters, increase awareness within healthcare settings, and shift organisational culture and practices [33]. This may also help increase a patient's motivation to adhere to treatment by establishing improved therapeutic alliance and respect [38]. Additionally, targeted education for not only care providers but also patients, families and communities may have value in combating stigma and humanising the treatment process. Social support (e.g., family or romantic partner) is a proven facilitator for improving patient engagement, retention and/or completion of SUD treatment [39, 40], highlighting the importance of educating and engaging social support entities across the SUD treatment continuum among individuals with StUD. Peer support specialists and/or case managers may be beneficial in assisting patients in navigating treatment and serving as a bridge to critical support systems, thereby enhancing treatment and recovery outcomes, by overcoming patient barriers and advocating at the societal level for individual needs (e.g., housing; [41]).

Findings also indicated a need for community‐level education and resource sharing to combat stigma. Community stigma can contribute to structural stigma, which in turn can influence local and institutional policies and cultural norms [42], creating barriers that limit the availability and accessibility of medication treatment options for StUD. Health communication campaigns are effective at reducing public health stigma by disseminating information and education. For example, the Communities That Heal intervention, a facet of the HEALing Communities Study that aims to reduce overdose fatalities across 67 communities in four US states, has reduced stigma toward people treated for OUD among its participants (i.e., individuals in participating communities) over time. This success is attributed to educating community members about OUD and evidence‐based treatments, and co‐creating communication campaigns with community coalitions [42].

Our findings also highlight the potential benefits of care providers and SUD treatment settings using a patient‐centred approach to StUD treatment to improve the clinical encounter. Patient‐centred and individualised SUD treatment approaches have been shown to increase patient service use and offer opportunities for care providers to educate and involve patients in care decision‐making [43, 44, 45]. However, a recent survey of a nationally representative sample of 657 SUD clinics in the United States found that only 23% of clinics were utilising patient‐centred approaches to increase patient engagement [45]. StUD is complex and highly individualistic and may require multimodal treatment plans [9]. For example, research has recommended that medication for StUD treatment would benefit from being paired with behavioural therapies for maximum effectiveness [46, 47], a finding supported by the current study, indicating the need for a holistic approach to StUD treatment. Additionally, combinations of medications may be more effective than individual pharmacotherapies [48], given patients' desire for medications that can address other symptoms of withdrawal and/or comorbidities, including depression, attention‐deficit/hyperactivity disorder, sleep problems or pain [49]. This is also in line with the current study's findings, with patients reporting unmet mental or physical health needs being a significant barrier to StUD treatment engagement.

Other significant societal‐level barriers that also highlight the significance of a holistic approach to StUD treatment access include cost, lack of reliable transportation, and physical location (e.g., rurality). Considering social determinants of health at the patient, treatment organisation, healthcare system, and societal levels that influence equitable service provision is critical for treatment engagement and adherence [38, 50]. More specifically, a significant portion of our patient and care team samples resided in Texas, a non‐Medicaid expansion state, and noted that social determinants, coupled with limited coverage options, can significantly affect treatment access. These findings underscore the importance of societal and outer context factors, including state policy environments, in shaping readiness for adoption of medications for StUD. At the organisation level, clinics that treat individuals with StUD would benefit from considering patient‐ and system‐level challenges and how they may impact treatment outcomes. Case managers and/or peer support specialists may assist with care coordination and addressing social determinants of health, such as securing housing, employment or insurance. Care team members with lived experience may be well‐positioned to engage patients with StUD and assist in developing care plans that better address individual needs [25].

It is useful to consider these findings in terms of issues specific to StUD versus SUD, more broadly. Barriers such as limited treatment access and continuity, provider capacity and care coordination, lack of patient‐centred care or supports (e.g., transportation), and provider and community stigma related to medication treatment are documented across several SUD types and care settings [38, 45, 50, 51]. However, many of the findings we report here seemed to be uniquely tied to stimulant use and StUD; for example, multilevel stigma, lack of FDA‐approved pharmacotherapies, and concerns regarding off‐label medication use. This suggests that some barriers to medication for StUD may be unique and require tailored solutions, including at the policy and systems levels. Expanding access to medications for StUD may require organisations and/or healthcare systems to invest in additional infrastructure (e.g., data systems), expand care teams (e.g., enhance case management), train staff and implement reimbursement mechanisms (e.g., value‐based payment) that support holistic care models inclusive of pharmacotherapies [21, 52, 53]. Fragmentation across funding streams and service delivery systems is a persistent barrier to SUD treatment engagement, access and continuity. The added layer of stigma associated with stimulant use and StUD may further exacerbate these disparities.

These multilevel contextual barriers to StUD treatment underscore the need for the development and use of multicomponent implementation strategies, the combination of two or more strategies, to address complex, multilevel barriers [54]. Implementation strategies are methods used to improve the adoption, implementation, or sustainability of an intervention [55]. Study findings can inform the development of strategies—for example, provider training curricula, resource toolkits (e.g., education materials for patients) and clinical guidelines, considerations for provider audit and feedback to support safe clinical decision‐making and increase use of patient‐centred care approaches, as well as the potential integration into existing care models (e.g., primary care or behavioural health) through external facilitation that supports clinics in collaborative problem‐solving and organisational change (e.g., polices and procedures) [56, 57, 58, 59]. Although there is an increasing body of evidence demonstrating the utility of multicomponent implementation strategies to increase delivery of MOUD [17, 18, 19], this is the first study of which we are aware that elicits patient or care team perspectives on readiness for and strategies to accelerate the adoption of medication for StUD. Over two decades of concerted efforts to implement and scale MOUD in real‐world practice have revealed significant and persistent challenges to achieving routine delivery of services. Therefore, as evidence of the efficacy and effectiveness of medication for StUD progresses, it is crucial to identify barriers to and strategise for implementation earlier along the translational pipeline to accelerate availability and systematic delivery.

### 4.1 Limitations

Although this study has several strengths, including its large sample size that allowed for a comprehensive examination of contextual factors likely to affect the adoption and implementation of medication for StUD, study participants were predominantly from Texas. Therefore, the generalisability of the current study's findings may be limited to settings with similar healthcare systems, SUD regulations, and insurance policies (e.g., Medicaid). Future research would benefit from the inclusion of more participants residing in regions and countries with differing regulations and practices. Additionally, we did not track participant demographics, including gender, race/ethnicity, age, level of education, or insurance status, due to the sensitive nature of the topic and efforts to minimise participant burden and protect confidentiality. Future research would benefit from the incorporation of patient and care team demographic factors and consideration of their potential impact on contextual factors and readiness for StUD medication treatment. Finally, although patient participants were confirmed to have a current or prior SUD diagnosis, we did not collect information on the type of diagnosis (i.e., StUD, OUD) or ask patients to identify a primary SUD, potentially affecting the interpretation and generalisability of study findings. However, patient recruitment primarily occurred through provider referral and/or SUD treatment settings, and recruitment materials explicitly outlined the study's focus on stimulant use and StUD.

## 5 Conclusions

Findings from the current study highlight multilevel contextual factors likely to facilitate or hinder the adoption of medication treatment for StUD. Both patients and care teams emphasised the need for holistic StUD treatment approaches that integrate medication with mental health, physical and social services and consider individual‐level challenges that may impact treatment access and engagement. Examining the different contextual levels where stigma may permeate is critical for identifying future strategies, including where education, training and/or awareness about StUD and its treatment may be required. Utilising implementation science frameworks that emphasise implementation determinants both within and outside of clinical practice settings can help identify evidence‐based strategies and develop complementary tools that may increase the likelihood of desired behaviour change and/or intervention outcomes [54]. Examining contextual factors that may impact the StUD treatment landscape can assist in closing the research‐to‐practice gap, as the clinical efficacy and effectiveness of medications for StUD continue to evolve globally.

## Author Contributions

Each author certifies that their contribution to this work meets the standards of the International Committee of Medical Journal Editors.

## Funding

This work was supported by the National Center for Advancing Translational Sciences (1UM1TR004538‐01) (Dr Robert Clark, PI). The content is solely the responsibility of the authors and does not necessarily represent the official views of the National Institutes of Health or National Center for Advancing Translational Sciences.

## Conflicts of Interest

The authors declare no conflicts of interest.

## Data Availability

The data that support the findings of this study are available from the corresponding author upon reasonable request.
